# The role of microvascular endothelial WNT signaling the formation of the blood brain barrier

**DOI:** 10.1186/2193-1801-4-S1-L47

**Published:** 2015-06-12

**Authors:** Maria Grazia Lampugnani, Luca Bravi, Elisabetta Dejana

**Affiliations:** IFOM, Italy

**Keywords:** beta-catenin, Cerebral Cavernous Malformation, endothelium

We analyzed the pathological consequences of abnormal Wnt/β-catenin signaling in endothelial cells of brain vessels using a murine model of Cerebral Cavernous Malformation (CCM) disease that develops after endothelial-cell-selective ablation of the CCM3 gene. We report increased transcription activity of β-catenin in CCM3-knockout endothelial cells in in-vitro and in-vivo models. Such activation is cell-autonomous, independent of Wnt-receptor stimulation, does not induce canonical Wnt/β-catenin signaling and represents an early response to CCM3 ablation that initiates the expression of EndMT makers before the onset of Tgf-β/BMP signaling which is required for the progression of the pathology, as we have previously described. We also show that the NSAIDs sulindac sulfide and sulindac sulfone, which attenuate β-catenin transcription activity, significantly reduce the number and dimension of vascular lesions in the central nervous system of mice with endothelial cell CCM3 knockout. These NSAIDs thus represent pharmacological tools for inhibition of the formation of vascular lesions, particularly with a view to patients affected by the genetic variant of CCM, who continue to develop new malformations over time.

